# A Newly Emerged Cutaneous Leishmaniasis Focus in Northern Israel and Two New Reservoir Hosts of *Leishmania major*

**DOI:** 10.1371/journal.pntd.0002058

**Published:** 2013-02-21

**Authors:** Roy Faiman, Ibrahim Abbasi, Charles Jaffe, Yoav Motro, Abdelmagid Nasereddin, Lionel F. Schnur, Moshe Torem, Francine Pratlong, Jean-Pierre Dedet, Alon Warburg

**Affiliations:** 1 Department of Microbiology and Molecular Genetics, The Institute for Medical Research Israel-Canada, The Kuvin Centre for the Study of Infectious and Tropical Diseases, The Hebrew University - Hadassah Medical School, Jerusalem, Israel; 2 Department of Vertebrates and Snails, Plant Protection and Inspection Services, Ministry of Agriculture and Rural Development, Bet-Dagan, Israel; 3 Kibbutz Sde Eliyahu, M. P. Beit She'an, Israel; 4 Université de Montpellier 1, Centre National de référence des Leishmania, UMR MIVEGEC (UM1, CNRS 5290, IRD224), Laboratoire de Parasitologie-Mycologie, CHU de Montpellier, Montpellier, France; National Institutes of Health, United States of America

## Abstract

In 2006/7, 18 cases of cutaneous leishmaniasis (CL) were reported for the first time from Sde Eliyahu (pop. 650), a village in the Beit She'an valley of Israel. Between 2007–2011, a further 88 CL cases were diagnosed bringing the total to 106 (16.3% of the population of Sde Eliyahu). The majority of cases resided in the south-western part of the village along the perimeter fence. The causative parasite was identified as *Leishmania major* Yakimoff & Schokhor, 1914 (Kinetoplastida: Trypanosomatidae). *Phlebotomus papatasi* (Scopoli), 1786 (Diptera: Psychodidae) was found to be the most abundant phlebotomine species comprising 97% of the sand flies trapped inside the village, and an average of 7.9% of the females were positive for *Leishmania* ITS1 DNA. Parasite isolates from CL cases and a sand fly were characterized using several methods and shown to be *L. major*. During a comprehensive survey of rodents 164 Levant voles *Microtus guentheri* Danford & Alston, 1880 (Rodentia: Cricetidae) were captured in alfalfa fields bordering the village. Of these 27 (16.5%) tested positive for *Leishmania* ITS1 DNA and shown to be *L. major* by reverse line blotting. A very high percentage (58.3% - 21/36) of Tristram's jirds *Meriones tristrami* Thomas, 1892 (Rodentia: Muridae), found further away from the village also tested positive for ITS1 by PCR. Isolates of *L. major* were successfully cultured from the ear of a wild jird found positive by ITS1 PCR. Although none of the wild PCR-positive voles exhibited external pathology, laboratory-reared voles that were infected by intradermal *L. major* inoculation, developed patent lesions and sand flies became infected by feeding on the ears of these laboratory-infected voles. This is the first report implicating *M. guentheri* and *M. tristrami* as reservoirs of *Leishmania*. The widespread co-distribution of *M. guentheri* and *P. papatasi*, suggests a significant threat from the spread of CL caused by *L. major* in the Middle East, central Asia and southern Europe.

## Author Summary

This study dealt with the ecological and epidemiological characterization of a newly emerged dermal disease caused by *Leishmania* parasites transmitted by the bite of infected sand flies. In the first five years of its emergence, the human infection rate in Kibbutz Sde Eliyahu, Israel reached 16%. The majority of cases resided along the periphery of the Kibbutz. To identify the vector species, we performed systematic trapping of sand flies in different habitats inside and outside the inhabited areas. Sand flies were identified in the laboratory and shown to comprise mainly *Phlebotomus papatasi*. Since the causative agent of human cases was shown to be *L. major*, we examined sand flies for *Leishmania* infections by PCR and found a high infection rate amongst sand flies trapped near houses in late summer (11.8%). To confirm the *Leishmania* species, we isolated live parasites from the gut of a wild-caught sand fly female. The cultured isolate was identified as *L. major* by several methods. The Levant vole, abundant in the surrounding fields and Tristram's jird found further away from the Kibbutz were tested for infection rate and their capacity for hosting the parasite and were both incriminated as new possible hosts.

## Introduction

The leishmaniases are parasitic diseases caused by *Leishmania* parasites and transmitted by phlebotomine sand flies in tropical, subtropical and temperate regions of some 98 countries [1], [2]. Three species of *Leishmania* cause leishmaniasis in Israel. Infections with *L. major* and *L. tropica* Wright, 1903 cause cutaneous leishmaniasis (CL) while *L. infantum* Nicolle, 1908 causes mainly canine but also human visceral leishmaniasis (VL) [3]. Recent studies have clearly documented the rapid geographical expansion and steep increase in the number of CL cases caused by *L. tropica* in northern Israel and the Palestinian West Bank [4], [5], [6]. Similarly, the incidence and geographic distribution of dogs with VL is clearly on the increase [7], [8]. On the other hand, *L. major* foci remained geographically stable with case numbers being directly proportional to the number of persons encroaching upon the zoonotic foci in the deserts of southern Israel [3], [9].

In 2007 physicians at the dermatology department of Ha'emek Hospital in Afula, northern Israel began to see increasing numbers of CL cases among residents of the Beit She'an Valley, north-eastern Israel. Skin scrapings from lesions were sent for diagnosis to the Hebrew University – Hadassah Medical School where the causative parasites were identified as *L. major* using ITS1 PCR and subsequent restriction fragment length polymorphism (RFLP) analysis [10], [11]. Concurrently, residents of Kibbutz Sde Eliyahu (Beit She'an valley) approached us for assistance in controlling sand flies.

*L. major* in Israel was traditionally restricted to desert foci where the parasites infect Sand rats *Psammomys obesus* Cretzschmar, 1828 (Rodentia: Muridae) and Sundevall's jirds *Meriones crassus* Sundevall, 1842 (Rodentia: Muridae), the chief reservoir hosts in the region [9], [12], [13], [14], [15], [16]. *P. papatasi* is the proven vector of *L. major* throughout much of its distribution including Israel [9], [16], [17]. However, preliminary studies in Sde Eliyahu made it clear that this emerging *L. major* focus was ecologically distinct; the village of Sde Eliyahu is surrounded by agricultural fields not desert. Moreover, it is located north of the recognized geographic range of both *P. obesus* and *M. crassus* which inhabit wild desert regions characterized by sandy or loess soils and chenopod bushes ([18] and G.Shenbrot, pers. comm.).

Here we report on findings from a comprehensive study conducted over five years in Kibbutz Sde Eliyahu in which we decipher the transmission cycles, incriminate the vector and implicate two new reservoir hosts of *L. major*. Our findings suggest the likelihood of CL caused by *L. major* spreading to new areas in Israel, neighboring countries and perhaps northwards into south European countries and central Asia.

## Methods

### Ethics

Medical records: Patients all sought medical treatment for CL and were not enrolled in a study. We received geographical data on where patients live and anonymous diagnostic data were collected in the lab to be correlated with the geographical data. Private medical records were not accessed in this study nor were names or other identifying aspects provided. The Helsinki Committee on Research Involving Human Subjects of The Hebrew University – Hadassah Medical School of Jerusalem, Israel approved the study on “Emergence of Cutaneous Leishmaniasis in the Middle East: An Investigation of *Leishmania tropica*” under which medical data were obtained (permit No. 362-7.09.07).

Trapping of wild sand flies and rodents and experimentation on rodents was approved by the Israel Nature and Parks Authority (INPA permit No. 2009/32202), under the wild life protection act of 1955, and the national parks protection and memorial sites act of 1998 (www.parks.org.il/).

The INPA permit allowed for trapping of 1000 voles and 300 jirds on the condition they would be sacrificed or otherwise kept in captivity when experimentation was over; anesthetization was to be carried out by intraperitoneal injection of ketamine and xylazine according to specimen weight. Euthanization would follow the ketamine/xylazine overdose route.

### Study site

All field work was conducted in or near Sde Eliyahu (32°26′N, 35°30′E, Alt. −185 m. Pop. 650), a collective agricultural settlement ( = Kibbutz) located 6 km south of the town of Beit She'an, in the northern Jordan Valley of Israel. The climate is hot and dry; the mean maximal daily temperature in summer is 35°–37°C and 18°–22°C in winter. Average annual rainfall is 280 mm. Farmers in Sde Eliyahu practice mostly organic farming, utilizing very few fertilizers or pesticides (www.seliyahu.org.il/eOrganic.htm).

### Human cases of Cutaneous Leishmaniasis

**Figure 1:**
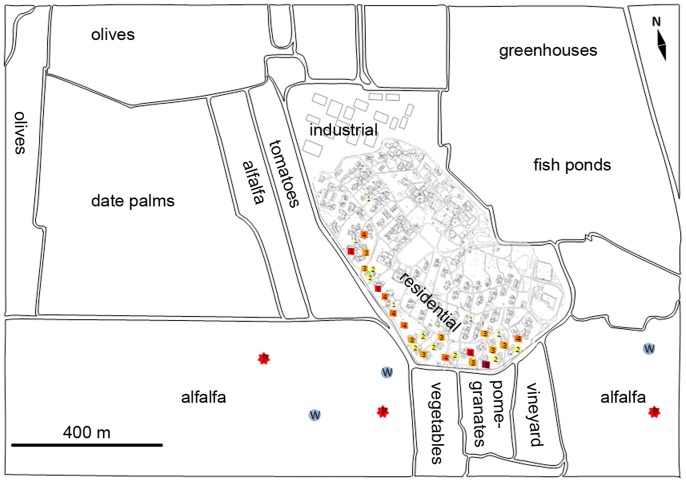
Annotated map of Sde Eliyahu and vicinity showing inhabited areas of the Kibbutz and the surrounding agricultural areas. Houses where CL cases reside are marked with colored squares. The number of cases appears in each square and a darker color denotes more cases. The approximate location of different cultivars is marked and type of cultivar noted. The letter **P** in a red star denotes alfalfa fields where voles were captured by plowing, while a blue circle containing a **W** denotes the approximate location where voles were captured by flooding.

The diagnostic data of all CL cases from Sde Eliyahu that were registered in either the local clinic or regional hospital (HaEmek Hospital, Afula), were used to associate between the season and locality of morbidity. This list included the year of birth, date of positive diagnosis, diagnostic test conducted and diagnosing facility. Personal details were withheld, and locations of their residences were mapped anonymously on a blank map of the site (Fig. 1).

### Trapping and identification of sand flies and rodents

Sand flies: Sand flies were trapped using CO_2_ (dry ice or compressed gas) - baited miniature CDC - type traps assembled in our lab [19]. Sand fly trapping within the Kibbutz was conducted after approval of the local household inhabitants. Trapping in the fields was given consent by the Kibbutz farming secretariat. Sand flies were transported to the laboratory where they were counted, sexed and preserved in 100% ethanol, frozen (−20°C) for PCR or dissected fresh for parasite isolation. Methods for mounting and identification were described previously [19].

Rodents: Voles that do not usually enter rodent traps were captured by hand after being expelled from their burrows by flooding with water or by plowing. Several active burrow systems in alfalfa fields located 30–300 m from the peripheral houses of the village were selected for flooding (Fig. 1: marked with W in blue). A 1000 L mobile water tank with a 20 m long hose was towed by tractor between the burrows in alfalfa fields. Each tank-full enabled the flooding of 3–4 burrow systems (5–8 exit holes). Voles escaping from flooded burrows were captured by hand wearing protective gloves. In advance of scheduled plowing of alfalfa fields we were notified and arrived a day in advance. We walked behind the tractor-drawn plow and captured voles that emerged from their destroyed burrows. Plowing was conducted in three alfalfa fields up to 400 m away from the village fence (Fig. 1; marked with P in red stars).

Other rodents (e.g. jirds and mice) were trapped with live rodent traps (*Sherman*, H.B. Sherman Traps. Tallahassee, FL, USA). Traps were dispersed 15–20 m apart, baited with a peanut snack (*Bamba*, Osem Ltd. Holon, Israel) attractive to many rodents [20].

### *Leishmania* isolates and DNA

Human cases of CL: The following procedure was performed routinely by a certified physician or nurse on suspected CL patients referred by their Health Maintenance Organizations (HMO) for diagnosis at HaEmek or Hadassah hospitals. The skin was sterilized and the exudates from superficial skin scraping of the margins of the lesion were blotted on to sterile filter paper [21]. In three cases, *Leishmania* isolates were obtained by seeding lesion exudates mixed with sterile PBS into NNN slants overlaid with M199 medium supplemented with 15% fetal calf serum (FCS) containing penicillin (100 IU/mL), streptomycin (100 µg/mL), pH 7.4 [22]. Identification of the causative agent of the disease was performed at the department of microbiology and molecular genetics in The Hebrew University of Jerusalem, using molecular tools (ITS1 PCR followed by RFLP) described below.

Sand flies: For parasite isolation, sand fly females were anesthetized, placed in a tea strainer and washed in 5% detergent solution. They were rinsed in sterile water, dipped briefly in 70% ethanol and transferred to sterile PBS supplemented with penicillin (100 IU/mL), streptomycin (100 µg/mL). Their guts were dissected on sterilized glass slides, using flame-disinfected forceps and examined for the presence of promastigotes under a phase-contrast microscope with a 40× objective. The guts of promastigote-infected sand flies were seeded into NNN slants (see above) further supplemented with 5-fluorocytosine (1,500 µg/ml, Sigma) to suppress fungal growth and 2% sterile human urine to enhance promastigote growth [23].

Rodents: Field-caught rodents were anaesthetized using a ketamine/xylazine mixture. Their ears were scrubbed with detergent solution (10%) followed by ethanol (70%). Small skin snips from the ear pinnae were mashed onto filter paper (Whatman, 3MM) for PCR and/or seeded into NNN slants (see above) for parasite isolation. For monitoring infection in lab-reared voles and jirds superficial skin scraping was performed using sterile blood lancets and the blood/exudate droplets were blotted onto pre-marked filter papers and microscope slides. Filter papers and glass slides were allowed to air-dry and kept at room temperature until use. Procedures were performed under permit of the Israel Nature and Parks Authority (INPA, permit No. 2009/32202).

### Identification of parasite isolates

Molecular biological (ITS1/RFLP) [10] and serological (EF sero-typing) [24] characterization of *Leishmania* isolates were performed at the Department of Microbiology and Molecular genetics, The Hebrew University-Hadassah Medical School, Jerusalem. Biochemical characterization using isozyme electrophoreses was done at the Centre National de référence des *Leishmania*, Université Montpellier 1, Montpellier, France [25].

### DNA extraction

Samples were excised from the filter papers using a scalpel or a paper punch. For samples from humans and rodents, DNA extraction was performed using the phenol-chloroform procedure described in detail previously [21]. Sand fly DNA extraction was performed using potassium acetate as described elsewhere [26].

### Polymerase Chain Reaction (PCR) and RFLP analysis of the ITS1 PCR amplicon

*Leishmania*-specific ribosomal Internal Transcribed Spacer 1 (ITS1) gene was amplified using the primers LITSR (5-CTG GAT CAT TTT CCG ATG-3) and L5.8S (5-TGA TAC CAC TTA TCG CAC TT-3) [10]. *L. major* MHOM/IL/1967/Jericho II was used as a positive control, after DNA was extracted from cultured parasites.

PCR products (8 to 20 µl) were digested with BsuRI (MBI Fermentas), a *Hae III* prototype, according to the manufacturer's instructions, and the restriction fragments were analyzed by gel electrophoresis at 120 V in 1 X Tris-acetate-EDTA buffer in 2.5% agarose gels (FMC BioProducts, Rockland, ME). The fragments were visualized by UV light [10], [21].

### Reverse-line blotting

*Leishmania* species specific ITS1 oligonucleotide probes were designed and hybridization procedures followed those previously described [27]. Briefly, 5′-amino modified oligonucleotides were immobilized on Biodyn C membrane strips (Pall Biomedical, USA). Strips were incubated in pre-hybridization solution (2× SSC, 0.1% SDS) for 30 minutes at 46°C with gentle shaking followed by hybridization with Biotinylated *Leishmania* ITS PCR product at 46°C for 1 hour. The membrane strips were washed with 0.7× SSC, 0.1% SDS for 20 minutes. The Hybridized biotinylated amplicon was detected by incubating the strips in strepavidin-HRP (diluted in 2× SSC, 0.1% SDS) for 30 minutes at room temperature and color development after the addition of 0.1 mg/ml of 3,3′,5,5′ tetramethylbenzidine (TMB) (Sigma), dissolved in 0.003% H_2_O_2_ in 0.1 M sodium citrate (pH 5.0).

### Laboratory infection of sand flies

*Phlebotomus papatasi* sand flies originating in Sde Eliyahu were reared according to the method of Modi and Tesh (1983) [28]. Five-day-old females (F1) were fed through chick-skin membranes on heparinized rabbit blood containing 5×10^6^/ml stationary-phase *L. major* promastigotes (IPAP/IL/2010/LRC-1475) isolated from a sand fly caught in Sde Eliyahu. Blood-fed flies were starved for 24 h and thereafter, maintained on a 50% aqueous honey (wild flowers) solution, at 26°C and 65% relative humidity. Blood-engorged females were sacrificed for determining infection 6–8 days post blood feeding. Parasite density was graded according to accepted criteria [29].

In an attempt to perform xenodiagnosis on voles and jirds, 5-day-old *P. papatasi* females were starved for 24 h and allowed to feed on anesthetized wild-caught rodents that were found positive for *Leishmania* by ITS1 PCR or infected artificially. The infected skin was exposed to blood-questing females inside a 20 ml glass vial covered by a tight cotton-mesh. Feeding was allowed for the duration of the anesthesia (approximately 45–60 min) in a darkened room at 24°C.

### Laboratory infection of voles

The experimental procedures were approved by the Israel Nature and Parks Authority (INPA permit No. 2009/32202). The voles used in this study were F2 progeny of wild-caught voles from Sde Eliyahu that were reared at the Israel Ministry of Agriculture rodent quarantine facility (PPIS) at Beit Dagan. Prior to all experimental procedures, voles were anesthetized intraperitoneally with ketamine (10%, 50 µl) and xylazine (2%, 10 µl). Animals were inoculated intradermally in the ear pinnae, above the snout, and in hind foot pads, using 10^6^/ml stationary-phase *L. major* promastigotes (Sde Eliyahu sand fly derived strain IPAP/IL/2010/LRC-1475), in 20 µl M-199 culture medium using a 29G needle. Blood-spot and smear biopsies for PCR diagnosis were taken weekly from the inoculation sites, beginning one week post inoculation (P.I).

## Results

From 2007 to 2011, 106 cases of CL from Sde Eliyahu were recorded. The majority (84.0%) were persons residing in peripheral houses close to the southern and south-western borders of the kibbutz (Fig. 1, Table 1). The first three cases were diagnosed in 2006 followed by 15 in 2007, and the peak of the outbreak that occurred in 2008 with 50 (reported) CL cases in Sde Eliyahu. In 2009, 2010 and 2011 case incidence dropped to 11, 9 and 18, respectively. The case incidence in Sde Eliyahu included almost all the residents of the peripheral houses (Fig. 1) and circa 16% of the entire Kibbutz population (106/650).

**Table 1:** The yearly distribution of CL cases from Sde Eliyahu.

On perimeter	Total Cases	Year
16(88.8%)	18	2006/7
46(92%)	50	2008
6(54.5%)	11	2009
8(88.9%)	9	2010
13(72.2%)	18	2011
89(84.0%)	106	Total

Total numbers of cases are shown on the left column and those residing adjacent to the perimeter fence are shown on the right column.

### *Leishmania* parasites

**Figure 2:**
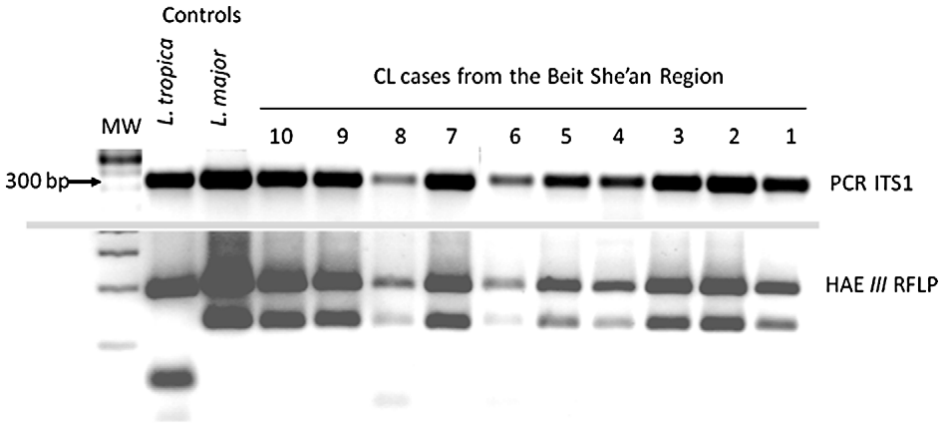
ITS1 PCR gel electrophoresis of DNA from lesion biopsies of ten cases of CL from Sde Eliyahu. Top panel 300 bp, *Leishmania* ITS1 PCR product. Bottom panel shows the HAE*III* restriction fragments of the ITS1 PCR product. Note the double-bands, identical to the pattern of *L. major* and very different from the *L. tropica* control.

Of the 71 CL cases from the Beit She'an Valley diagnosed in the Hebrew University lab during the years 2006–2011, 56 were positive for *Leishmania* DNA by ITS1 PCR. Of these 51 were characterized by RFLP as *L. major* species (Fig. 2, Table 2). Five *L. major* isolates; three from human CL cases, one from a naturally infected jird and one from a naturally infected *P. papatasi* female sand fly captured near Sde Eliyahu, were also characterized using isoenzyme electrophoresis and excreted factor (EF) serotyping (Table 3). All isolates' ITS1 gene were sequenced and shown to be *L. major* (99.9–100%).

**Table 2:** Diagnosis of CL cases from the Beit-She'an Valley (2007–2011).

Location	Clinical examination	ITS1/PCR+	HAE*III* - RFLP		Not determined*
			*L. major*	*L. tropica*	
Sde Eliyahu	42	33	30	0	12
Beit She'an	16	11	9	1#	6
Kfar Ruppin	8	7	7	0	1
Tirat Tzvi	5	5	5	0	0
Total	71	56	51	1	19

* Parasites from these lesions were identified by ITS1 PCR as *Leishmania* but the species could not be identified, either because there was not enough DNA in the samples or because RFLP analyses were inconclusive.

# The causative parasites in all but one of the cases were determined as *L. major*.

**Table 3:** *Leishmania major* isolates from Sde Eliyahu.

Species#	WHO code	Source	Geograhpic location	EF serotype^1^	Zymodeme^2^
*L. major*	MHOM/IL/2008/LRC-L1338	human CL	Sde Eliyahu	A_1_	MON-103
*L. major*	MHOM/IL/2010/LRC-L1484	human CL	Bet She'an	A_1_B_2_	MON-103
*L. major*	IPAP/IL/2010/LRC-1475	*Phlebotomus papatasi*	Sde Eliyahu	A_4_	MON-103
*L. major*	MHOM/IL 2008/LRC-L1350;	human CL	Bet She'an	A_1_	MON-26
*L. major*	MMER/IL/2012/LRC-L1630	*Meriones tristrami*	Sde Eliyahu	A_1_	MON-103

# Species determined by DNA sequencing of the ITS1 PCR product.

1 EF serotype was determined according to [24].

2 Zymodeme profiles were determined according to [25], [54].

### Sand flies

**Figure 3:**
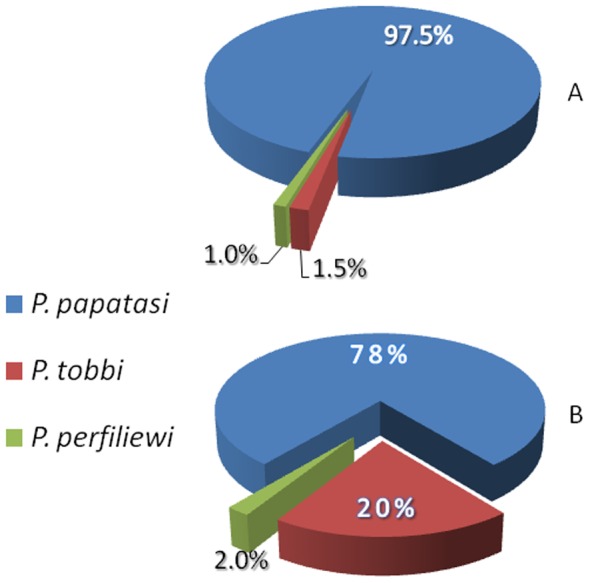
The species composition of phlebotomine sand flies captured using CO_2_ baited traps. A – Sample of sand flies trapped near the houses (n = 385) comprising 97.5% *P. papatasi*. B – Sample of sand flies trapped in an alfalfa field (n = 250) 1 km south of Kibbutz fence.

Routine sand fly monitoring was conducted from early July to late October 2009 (21 nights X 35 traps = 735 trap/nights). In total 3,087 females were trapped along the Kibbutz' peripheral fence and near houses and in an alfalfa field south-east of the Kibbutz (Fig. 1). A sample of 385 sand flies captured near houses and 250 sand flies from the field were identified showing that *P. papatasi* was by far the predominant species both near houses (97.5%) and in the fields (78%). *P.(Larrousius) tobbi* Adler & Theodor, 1930 and *P.(L.) perfiliewi* Parrot, 1930, neither of which is known to transmit *L. major*, made up the balance (Fig. 3). A sample of 1,047 (34%) *P. papatasi* females, trapped throughout the 2009 sand fly season next to the houses, were used to screen for *Leishmania* infections by ITS1 PCR. An average of 7.9% of the females was positive for *Leishmania* ITS1 DNA. The infection rate increased from 5.2% in August, peaking at 11.76% by late October (Table 4). Sand fly numbers decreased significantly in November as the sand fly season ended. A sample of ten ITS1 PCR products from wild caught sand flies was analyzed by reverse line blotting (RLB) and DNA sequencing to identify the *Leishmania* species. All samples were shown to be *L. major* (data not shown).

**Table 4:** *Leishmania* infection rate in *Phlebotomus papatasi* females throughout the sand fly season of 2009 in Sde Eliyahu.

Month	No. of sand flies tested	Positive^1^	Infection rate (%)
August	880	46	5.23
September	133	9	6.77
October	34	4	11.76

1 Infections were determined by ITS1-PCR.

### Rodents

In total 164 voles (*M. guentheri*) were captured by flooding burrows or by plowing of alfalfa fields (approximate locations marked with blue circled ‘W’ in Fig. 1). Thirty six (36) Tristram's Jirds (*M. tristrami*), were trapped using Sherman traps. Although the infection rates in jirds were high, their overall numbers were lower than voles and they were not found in proximity to the houses. Twenty-five of 36 were trapped in the olive grove (800–1000 m W of the nearest house), 9 in an alfalfa field (1 km E of the houses) and two were trapped in the date palm plantation (700 m NW of the houses) (Fig. 1). Conversely, voles were plentiful near the fence and the highest infection rates were recorded in the two fields closest to the Kibbutz (marked with red stars in Fig. 1).

**Figure 4:**
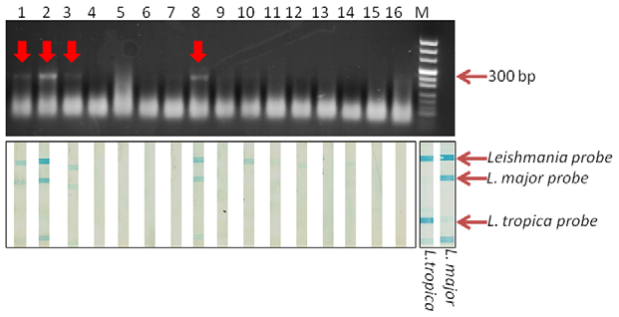
Detection and analyses of natural *Leishmania* infections in wild-caught voles. Top Panel: Detection of *Leishmania* DNA by ITS1 PCR. Bottom Panel: Identification of PCR products by reverse-line blotting. PCR-positive samples (1,2,3 & 8) were identified as *L. major*.

Animals were anesthetized and examined for skin lesions. There were no obvious signs of disease in any of the animals of either species. Small skin biopsies from ears and snout were tested for *Leishmania* infection by ITS1 PCR. Twenty-seven (27/164 or 16.5%) voles were positive for *Leishmania* DNA, as were 58.3% (21/36) of the jirds (Table 5). Samples that yielded sufficient PCR products, were shown to be *L. major* using reverse-line blotting (Fig. 4). One of the infected jirds that was kept in the animal facility for six weeks, developed patent lesions in its ear pinnae. Biopsies from the lesions were seeded into NNN culture tubes. The promastigotes that grew in the culture were identified as *L. major* by sequencing the ITS1 PCR product and excreted factor serotyping (Table 3).

**Table 5:** Rodent population and *Leishmania* infection rate.

Date	Location	Rodent species^1^	Captured	ITS1+
Mar 2008 to Jan 2011	Alfalfa fields (see Fig. 1)	*Microtus guentheri*	164	27(16.5%)
Aug 2008 to Jul 2011	Fence of village, olive grove, date plantation	*Mus musculus*	44	1 (2.3%)
Jul to Oct 2011	Olive grove, date plantation	*Meriones tristrami*	36	21(58.3%)

1 Data includes a rodent survey conducted with the ministry of agriculture.

In addition, 44 house mice *Mus musculus* Linnaeus, 1758 (Rodentia: Muridae) were trapped in different locations in or near Sde Eliyahu. *Leishmania* DNA was detected by PCR in a single mouse trapped in an olive grove, some 1,500 m NW of the Kibbutz (Table 5). A very faint band corresponding to *Leishmania* ITS1 PCR product was observed on the gel. There was no discernible external pathology (data not shown).

### Laboratory infection of voles

**Figure 5:**
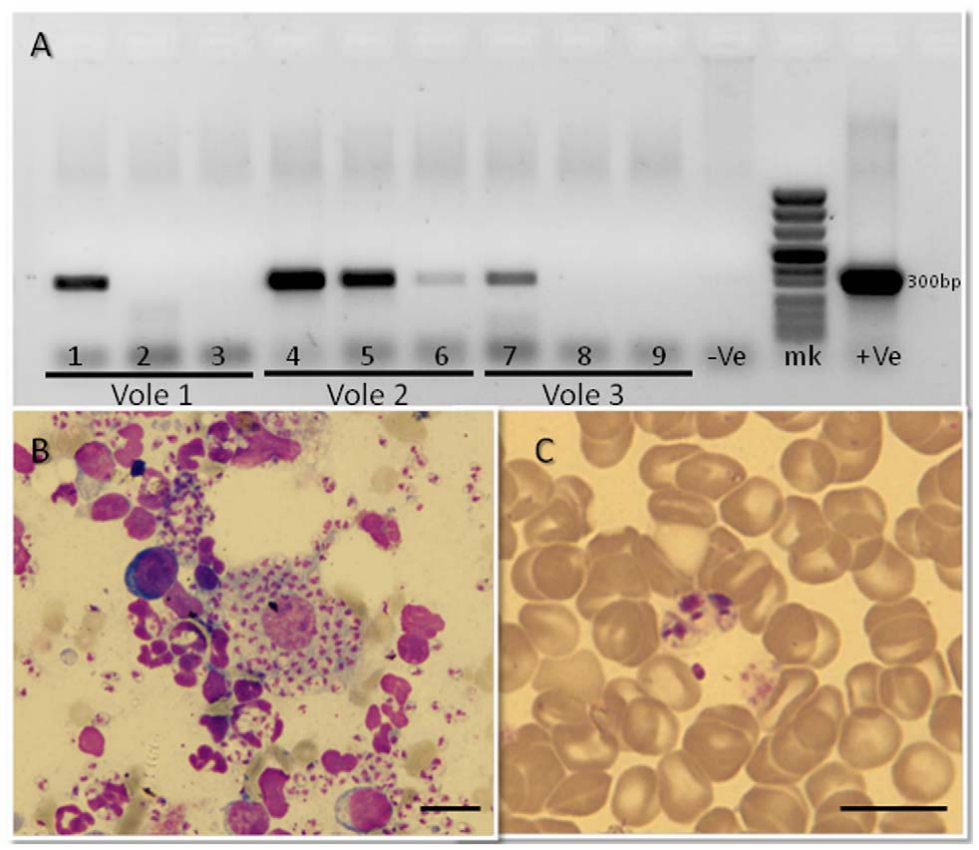
Artificial *L. major* infections in voles. (A) Leishmania-specific ITS1 PCR products (300 bp) in three voles, three weeks post-inoculation. Lanes 1,4,7 – ears. Lanes 2,5,8 – hind foot pads. Lanes 3,6,9 – snout. Biopsies were taken weekly after inoculation of 10^5^ late log -phase *L. major* promastigotes in each inoculation site. **−Ve** – negative control without DNA, **+Ve** – positive control containing *L. major* DNA, **mk** – marker reference ladder. (B) Micrograph of tissue smear showing amastigotes-laden macrophages from the ear of a vole, six weeks P.I. (C) Blood smear showing free amastigotes. Peripheral blood from the hind foot pad of an infected vole seven weeks P.I. Scale bars = 10 µm.

**Figure 6:**
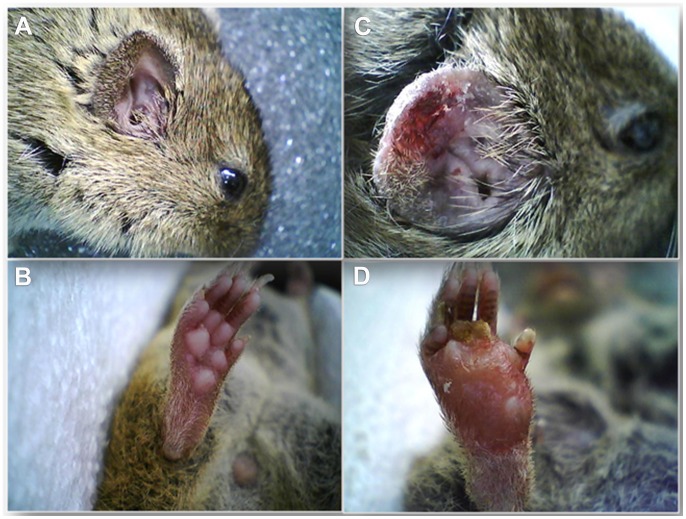
Pathogenesis of artificial *L. major* infections in voles' ears and hind foot-pads. Before infection (A, B), and 11 weeks post-infection with 10^5^ late log-phase *L. major* promastigotes (C, D). Infected ears (C) typically swell, depilate and ulcerate. Infected hind foot pads (D) were nodulated, ulcerated, became erythematous and edematous.

Three anesthetized voles were inoculated intradermally with 10^5^ late log-phase *L. major* promastigotes (Sde Eliyahu sand fly derived strain IPAP/IL/2010/LRC-1475). *Leishmania* DNA was detected by ITS1 PCR in all three voles beginning one week after inoculation and weekly henceforth (Fig. 5A). Infections flared up in the hind foot pads first (Fig. 6D) followed by the ears (Fig. 6C). No lesions developed in the snouts of any of these voles. The ear lesions exhibited pronounced disfiguring, swelling and hair loss (Fig. 6C). The hind foot pad lesions were distended and presented a dry yellowish ulceration (Fig. 6D). Giemsa-stained smears taken from infected ears and hind foot pads exhibited large numbers of amastigote-laden macrophages 4–5 weeks P.I onward (Fig. 5B).

### Laboratory infection of sand flies

Five-day-old laboratory-reared *P. papatasi* females (Sde Eliyahu colony) were fed through a chick-skin membrane on heparinized rabbit blood containing 2×10^6^ late-log phase *L. major* promastigotes/ml (strain IPAP/IL/2010/LRC-1475). Four females dissected three days after feeding had heavy infections in the thoracic midgut. The remaining ten females were left for seven more day to digest their blood meals and then to re-feed on anesthetized, healthy voles. This trial was repeated twice, however, females were reluctant to take a second blood meal and thus, artificial infections of voles by sand fly bite were not achieved.

To determine infectiousness of voles to sand flies, five day-old *P. papatasi* females that were starved for 24 hours were allowed to feed on the ear or footpad of an anesthetized vole that had been infected with *L. major* six weeks earlier. Fed females were separated and maintained in the insectary at 26°C on 50% aqueous honey solution. Females were sacrificed 6–11 days after feeding and their gut examined under phase-contrast illumination. In three repeats, a total of 20 *P. papatasi* females had fed and ten of these were infected. Most infected flies had heavy infections with promastigotes forming a plug in the thoracic midgut, stomodaeal valve area.

## Discussion

The outbreak in Sde-Eliyahu followed a pattern characteristic of CL caused by *L. major* resulting in a very high percentage (16.3%) of the village population, most of them residing along the perimeter, contracted the disease during the initial five years (Table 1). *P. papatasi*, the vector of *L. major* is highly anthropophilic (attracted to humans) and endophilic (enters and feeds inside houses) [30], [31], [32]. In contrast, *P. (Paraphlebotomus) sergenti* Parrot, 1917 that transmits CL caused by *L. tropica* in the region prefers animals and is much less endophilic [6], [33], [34]. Therefore, the efficiency of transmission is greater and the case incidence of CL in *L. major* foci is usually significantly higher than in *L. tropica* foci [3].

Concurrent with the studies reported here, we conducted a sand fly control trial by erecting a tall insecticide-treated barrier along the southern portion of the fence of Sde Eliyahu. The intervention markedly reduced the number of sand flies captured near houses and a significant relief in bite burden, was reported by the residents [19]. Moreover, many of the persons living on the periphery of Sde Eliyahu had already become infected by 2009 consequentially reducing the pool of susceptible individuals available for infection. Therefore, the causes for the decrease in CL cases noted in 2009 should be interpreted with caution.

The Sde Eliyahu focus is ecologically very different from the typical *L. major* foci throughout the wide geographical distribution of this parasite. Zoonotic *L. major* foci are normally found in desert or semi arid habitats where chenopod plants and other wild bushes flourish. *P. obesus*, the most important reservoir host, is highly fastidious, feeding on wild plants and burrowing under their roots [16], [35]. This rodent does not adapt well to highly modified agricultural environments [3], [9], [36], [37], [38]. Sde Eliyahu receives at least twice as much rain-fall annually than most other *L. major* foci and is surrounded by agricultural fields (Fig. 1). Significantly, both *P. obesus* and *M. crassus*, the known reservoir hosts of *L. major* in the Middle East were absent from Sde Eliyahu. On the other hand, innumerable burrows of voles were readily encountered in alfalfa fields, as well as orchards, vegetable gardens, date-palm plantations and olive groves bordering alfalfa fields surrounding the village [39]. Tristram's Jirds were not trapped in the immediate vicinity of the village only in orchards and olive groves some 1–2 km away [39], [40], [41]. *Leishmania* specific ITS1-PCR followed by RFLP showed that a high percentage of both rodent species (16.5% of the voles and 58.3% of the jirds) were exposed to *L. major* infections. In a PCR survey of wildlife conducted recently *M. tristrami* from south-western Israel were found infected with *L. major* in (8/59; 14%) [42]. Eight additional *Meriones* spp. have been positively incriminated as reservoir hosts of *L. major* in different parts of this parasite's distribution [13], [38], [43], [44], [45]. Therefore, finding *M. tristrami* naturally infected with *L. major* was not surprising.

The vole *M. guentheri* is abundant in agricultural areas [39] and geographically widespread, ranging from Israel's north and west through Syria and Lebanon to Turkey, Greece, Bulgaria and former Yugoslavia [46]. The species is also found eastwards all the way to Iran [47]. Abundant as the species may be, it had not been documented to harbor *Leishmania* parasites – possibly because of its abstention from entering traps [47]. In our studies we utilized alternative methods for capturing voles by plowing and flooding burrows. These techniques were highly effective but adult-biased since the adult voles were the ones captured escaping from disturbed burrows. In this respect, these approaches may have increased the chances of collecting *L. major* infected animals.

The ears of laboratory-infected voles were shown to be infectious to *P. papatasi* females. Ears are the preferred site for development of *L. major* in infected *P. obesus, M. tristrami, M. crassus* and apparently in voles as well. The sparse, short hair make ears accessible to probing sand flies, thus increasing chances of infection by bite. The inverse experiments, attempting to infect naïve voles by bite of artificially infected sand flies failed due to reluctance of the infected female *P. papatasi* to re-feed on the anesthetized voles. We were unsuccessful in obtaining live *L. major* cultures from wild-caught PCR-positive voles. Despite these somewhat disappointing results, we are reasonably confident in incriminating voles as the main *L. major* reservoir host in the Sde Eliyahu focus because, *M. guentheri* were abundant close to houses where CL patients lived and high proportions were PCR-positive for *L. major*. Moreover, lab experiments confirmed their susceptibility to local *L. major* (Figs. 5, 6) and infected voles were infectious to local *P. papatasi*. It is possible that *M. tristrami* served as bridge-hosts, facilitating the spread of *L. major* from *P. obesus*, naturally found in the Central Jordan Valley some 50 km to the south [17]. However, the sparse populations of *M. tristrami* found only in distant locations (>500 m) from houses' preclude this rodent from being regarded as the main direct reservoir host in the Sde Eliyahu focus.

Incriminating *P. papatasi* as the vector of *L. major* in Sde Eliyahu came as no surprise since this phlebotomine species is by far the most important vector of *L. major* throughout most of the very wide geographic distribution of this parasite [30], [37]. Moreover, the distribution of *P. papatasi* far exceeds that of *L. major* and extends northwards into Turkey, Greece and the Adriatic region, former Yugoslavia, the Balkans, Portugal and other south European countries and the Mediterranean littoral [48], [49], [50], [51], [52]. The absence of CL caused by *L. major* from these countries has hitherto been explained by the lack of suitable reservoir hosts. Our findings point to the possibility that *L. major*, having adapted to voles, will spread northwards into Turkey, central Asia and southern Europe where reservoir hosts and vector exist sympatrically.

Sde Eliyahu farmers practice mostly organic farming. Little-to-no rodenticides or insecticides are used and voles are controlled by encouraging nesting of barn-owls *Tyto alba* (Scopoli, 1769) (Strigiformes: Tytonidae) and Kestrels *Falco tinnunculus* Linnaeus, 1758 (Falconiformes: Falconidae) [41]. Sand flies are normally highly susceptible to insecticides [53]. Perhaps because insecticides are only used sparingly in Sde Eliyahu, sand flies were very abundant in agricultural fields and plantations (Fig. 3). It is possible that local organic farming practices combined with biological rodent control provided the setting for the intensive interaction of voles and sand flies, thereby, facilitating the adaptation of *L. major* to voles, and the emergence of CL in Sde Eliyahu. Notably, human CL cases have now spread to near-by agricultural communities, none of which practice organic farming.
